# Gibberellic acid induced parthenocarpic ‘Honeycrisp’ apples (*Malus domestica*) exhibit reduced ovary width and lower acidity

**DOI:** 10.1038/s41438-019-0124-8

**Published:** 2019-04-06

**Authors:** Kelsey D. Galimba, Daniel G. Bullock, Chris Dardick, Zhongchi Liu, Ann M. Callahan

**Affiliations:** 1 Appalachian Fruit Research Station, Agricultural Research Service, United States Department of Agriculture, 2217 Wiltshire Road, Kearneysville, WV 25430 USA; 2 Department of Cell Biology and Molecular Genetics, College of Mathematics and Natural Sciences, University of Maryland, College Park, MD 20742 USA

**Keywords:** Gibberellins, Fruiting, Plant molecular biology, Agricultural genetics

## Abstract

Fruit set and development are dependent on auxin, gibberellin, and cytokinin, which cause parthenocarpic development in many species when applied ectopically. Commercial sprays containing these hormones are used to improve apple fruit set, size, and shape, but have been implicated negatively in other aspects of fruit quality. We applied gibberellic acid (GA_3_), synthetic auxin (NAA), and the auxin-transport inhibitor NPA to ‘Honeycrisp’ apple flowers. Fruit retention and size were quantified throughout development, and seed number and fruit quality parameters were measured at maturity. GA_3_ alone caused the development of seedless parthenocarpic apples. At maturity, GA_3_-treated apples were narrower due to reduced ovary width, indicating that GA_3_ induced normal growth of the hypanthium, but not the ovary. GA_3_-treated fruits were also less acidic than hand-pollinated controls, but had similar firmness, starch, and sugar content. To further understand the regulation of parthenocarpy, we performed tissue-specific transcriptome analysis on GA_3_-treated, NAA-treated, and control fruits, at 18 days after treatment and again at maturity. Overall, transcriptome analysis showed GA_3_-treated and hand-pollinated fruits were highly similar in RNA expression profiles. Early expression differences in putative cell division, cytokinin degradation, and cell wall modification genes in GA_3_-treated ovaries correlated with the observed shape differences, while early expression differences in the acidity gene *Ma1* may be responsible for the changes in pH. Taken together, our results indicate that GA_3_ triggers the development of parthenocarpic apple fruit with morphological deviations that correlate with a number of candidate gene expression differences.

## Apples: Seedless ‘Honeycrisp’ fruit develop small cores with less acidity

Apple trees sprayed with a plant growth hormone called gibberellic acid develop seedless fruit that show morphological differences explained by changes in gene expression profiles. Ann Callahan from the US Department of Agriculture in Kearneysville, West Virginia, and colleagues showed that gibberellic acid induces flowers of the ‘Honeycrisp’ apple variety to develop fruit without fertilization, a process known as parthenocarpy. These fruit were seedless, with a thinner ovary at the fruit’s core and a lower acid content compared to normally pollinated controls. The researchers analyzed gene activity patterns 18 days after hormone treatment. They found differences in the expression levels of genes involved in cell division, hormone metabolism and cell wall dynamics, plus lower levels of an acidity-associated gene during early ovary development. The findings show that parthenocarpic Honeycrisp apples are possible, although they exhibit quality differences.

## Introduction

The angiosperm fruit is a structure derived from the ovary of the flower, which functions to protect and disperse the seeds. Prior to pollination, numerous genetic factors repress ovary development^1^, and as long as they are active, the ovary will senesce and no fruit will form^2^. This default repression pathway can be overridden by fertilization signals that trigger fruit set, the first stage of fruit development^3^. Current evidence supports the major role of three fertilization-induced hormones, auxin, gibberellin (GA), and cytokinin in the regulation of fruit set^4^. Individually, any of these hormones can stimulate parthenocarpic growth to some extent when applied ectopically; combined they can induce normal fruit growth even in the absence of fertilization^3^. Parthenocarpy is a condition in which the fruit develops independently of fertilization, and although this process is poorly understood, it is responsible for seedless cultivars of a number of commercially important fruit crop species, such as banana, eggplant, and fig^5^. In *Arabidopsis*, fertilization triggers an auxin response in the ovule, which upregulates the expression of GA biosynthesis genes in the ovule and causes GA to be translocated to the ovary wall, where it promotes cell expansion and differentiation^6,7^. Auxin also upregulates the production of cytokinin, and the two together contribute to cell division and fruit patterning^8,9^. While the *Arabidopsis* fruit is a dry fruit, hormone involvement in fruit development seems to be mostly conserved in other, fleshy-fruited species, such as grape, tomato, and strawberry^10–12^.

Apple is an emerging model system^13^, with a unique fruit morphology that makes it of particular interest in the study of fleshy fruit development. Apples are pomes, accessory fruits in which the ovary forms the relatively small non-fleshy core, and the attached cup-like hypanthium forms the fleshy tissue surrounding the ovary at maturity (Figure S1). A number of studies analyzing the role of hormones in apple fruit development have generally come to the consensus that, like other dry and fleshy fruits, apple development is dependent on the plant hormones gibberellin, auxin, and cytokinin^14–20^. When applied ectopically, these hormones have been shown to stimulate parthenocarpic development, increase fruit set, or improve fruit size and/or shape. This has led to their use as commercial sprays, specifically in conditions where fruit set or quality is expected to be low, such as following a critical freeze^21,22^.

Hormone applications designed to improve set and quality are not used to the extent as those designed for other purposes, like thinning or shifting harvest time. This is likely due to a number of inherent problems associated with these sprays: efficiency can differ from year to year, and results can vary widely between cultivars^23^. The biological cause of this variability is largely unknown, and incongruence between methodology and purpose in early hormone studies makes it difficult to draw conclusions regarding specific hormone effect. In addition, there are reports that indicate that ectopic hormone applications may have detrimental side effects, including advanced starch conversion, softer flesh, and less acidity, suggesting an acceleration in ripening^17,24^.

The aims of this study were to determine whether auxin and/or gibberellin could induce parthenocarpy, whether these hormones would create negative quality effects and which genes or genetic networks may be involved in these processes. We addressed these questions by applying exogenous hormones to flowers and then measuring fruit retention, size, quality, and gene expression. Retention and size measurements were used to assess induction of parthenocarpy. We analyzed size measurements in detail at maturity, taking into account the different organs making up the pome fruit in order to identify any effects on fruit shape. Other quality effects that have been reported previously^17,24^ were also analyzed. To determine the role of genes and genetic networks, we performed an RNA-seq analysis in an organ-specific manner. Better understanding of the effect that ectopic hormones have at the morphological and genetic level may give insight into how they mimic or bypass fertilization signals, how individual hormones function in this process, and how they may contribute to detrimental effects on final fruit quality.

## Results

Previous studies on the induction of parthenocarpic apple fruit suggested that auxin and bioactive gibberellins could be applied to flowers individually or in combination and result in seedless fruit. To test whether gibberellin and auxin can cause parthenocarpic ‘Honeycrisp’ apple development, we treated flowers with these hormones and then prevented pollination on these as well as a negative control (NC) to ensure developmental changes to the fruit were due to exogenous hormone treatment. Two positive controls: a hand-pollinated control, (HP) and an open-pollinated control, (OP) were also used to determine the normal levels of retention, size, seed production, and quality characteristics. We measured three signs of successful parthenocarpy, fruit retention, fruit enlargement, and absence of seed development, in addition to typical postharvest parameters to determine fruit quality.

### Fruit retention: auxin, gibberellin, and auxin combined with gibberellin cause similar rates of fruit retention throughout development

**Fig. 1:**
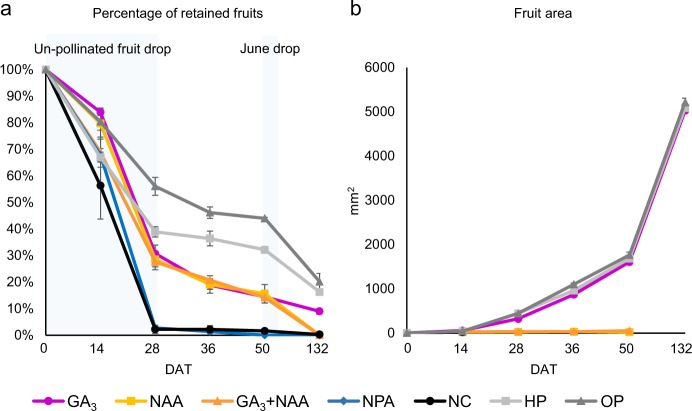
Fruit retention and size from treatment to harvest. **a** Average percentage of remaining fruit on each treated tree every 2 weeks, from initial treatment to 50 days after treatment (DAT), and at harvest once mature (132 DAT). Error bars indicate standard error among three replicates. Shaded regions indicate periods of fruit drop. At maturity, statistically less GA_3_-treated fruits were retained than HP or OP, but more than any other treatment. Statistical comparisons are reported in Table S1. **b** Average size of fruit present on each treated tree every 2 weeks, from 2 weeks until 50 DAT and at harvest once mature. Initial measurement (0 DAT) is estimated. At maturity, GA_3_-treated fruits were statistically similar in size to HP and OP. NAA and GA_3_ + NAA-treated trees had no fruit at time of harvest. NPA-treated and negative control (NC) trees had three and six fruits, respectively, all except one NC fruit had seeds. As these were likely missed in staging, size data were omitted past 14 DAT. Statistical comparisons are reported in Table S2. HP hand pollinated, OP open pollinated

Typically, only a small percentage of apple flowers will mature into fruits. Developing fruitlets are shed during two major periods of fruit drop; in the weeks following pollination, when un- or poorly-pollinated fruits are typically shed, and again a month or so later, during the so called “June drop”. To determine the effects of hormone treatments on fruit retention, we averaged the percentage of remaining fruit on each replicate every 2 weeks for the first 2 months (past “June drop”), and at maturity when fruit were harvested (Fig. 1a, Table S1). Initial total treated flower counts were: GA_3_ = 500, NAA = 420, GA_3_ + NAA = 484, NPA = 369, HP (hand-pollinated) = 312, OP (open pollinated) = 445, NC (negative control) = 431. All trees had shed some fruit by 14 days after treatment (DAT), with statistically similar percentages of retained fruit. All trees shed fruit in between 14 and 28 DAT, but at 28 DAT, average fruit retention percentages of hormone-treated trees (GA_3_-treated, NAA-treated, and GA_3_ + NAA-treated) and the HP control were similar, all higher than the NPA-treated or the NC, but lower than the OP control. At 36 and 50 DAT, all hormone-treated trees retained less fruit than either pollinated control. At the point of harvest, when fruits were mature, the GA_3_-treated tree retained on average 9% of its fruit. The NAA-treated, GA_3_ + NAA-treated, and NPA-treated trees dropped all fruit, and the NC had one remaining fruit. The GA_3_-treated tree also retained significantly less fruit than the HP and OP controls, which retained 16% and 20%, respectively. In summary, both GA_3_ and NAA, alone and in combination, caused the retention of un-pollinated fruit through the first 2 months of development. Only GA_3_ alone caused the retention of fruit through maturity. None of the hormone treatments were as efficient at causing fruit retention as pollination. The polar auxin-transport inhibitor NPA did not influence fruit retention, as fruit shed was equal to the un-pollinated NC fruit.

### Fruit size: gibberellin causes fruit to enlarge, but auxin and auxin combined with gibberellin do not

**Fig. 2:**
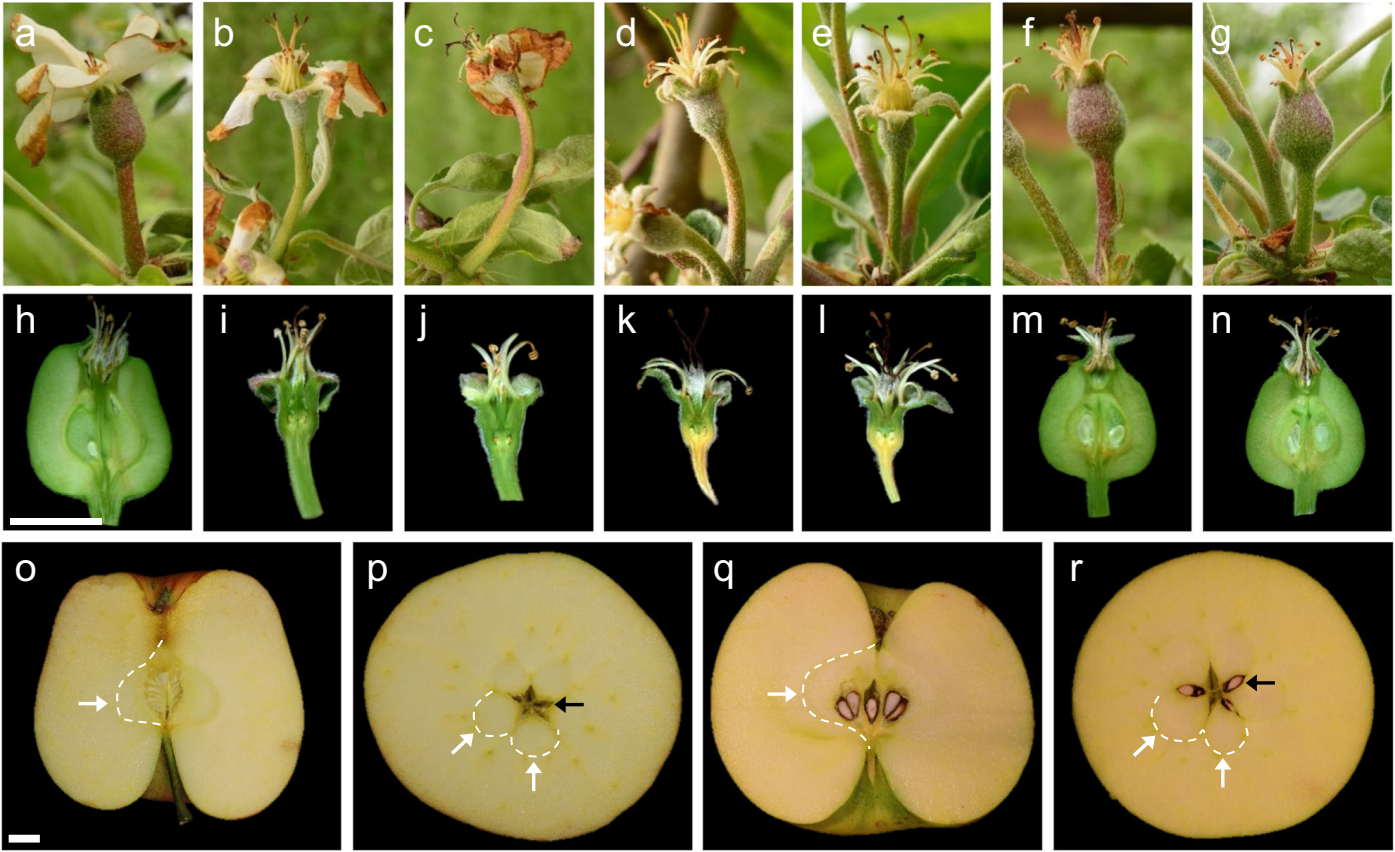
Fruit morphology of hormone-treated and control fruits. **a–g** Fruits on tree, 14 days after treatment (DAT). **a** GA_3_-treated. **b** NAA-treated. **c** GA_3_ + NAA-treated. **d** NPA-treated. **e** Negative control. **f** Hand-pollinated control. **g** Open-pollinated control. **h–n** Longitudinally sectioned fruits at 18 DAT. **h** GA_3_-treated. **i** NAA-treated. **j** GA_3_ + NAA-treated. **k** NPA-treated. **l** Negative control. **m** Hand-pollinated control. **n** Open-pollinated control. **o–r** Hand-pollinated and GA_3_-treated fruit morphology at harvest (132 DAT). **o** Longitudinal section of GA_3_-treated fruit. **p** Medial cross-section of GA_3_-treated fruit showing lack of seeds. **q** Longitudinal section of a hand-pollinated fruit. **r** Medial cross-section of hand-pollinated fruit showing seeds present in three out of five locules. Ovary wall/hypanthium boundaries partially indicated by white arrows and dashed line. Representative locules indicated by black arrows. Scale bar = 10 mm

To determine the effect of the different hormone treatments on fruit size, each fruit was measured from 14 to 132 DAT (Fig. 1b, Table S2). At 14 DAT, GA_3_-treated fruits were on average, significantly larger than all other treatments and controls. The other hormone-treated and NPA-treated fruits were significantly similar in size to the negative control, and all smaller than the pollinated controls at 14 DAT. Dissecting GA_3_-treated fruit at 18 DAT revealed ovules that were larger than NC, but smaller than HP or OP (Fig. 2h, l–n). Dissections of the other hormone-treated fruit at 18 DAT revealed that NAA and NAA + GA3-treated fruit had small, brown, and atrophied ovules (Fig. 2i, j). Both NPA-treated and NC fruit had small, green ovules, and the majority of fruit still remaining on these trees were yellow and soft, indicating that they were senescing (Fig. 2k, l). Both HP and OP controls had large fruits containing large, developed seeds (Fig. 2m, n). By 28 DAT, the NAA-treated and GA_3_ + NAA-treated fruits were significantly smaller than all others, and this relationship remained constant throughout development (Fig. 1b). GA_3_-treated fruits stayed similar in size to pollinated controls throughout development, including at maturity. There were very few NPA-treated and NC fruits retained after 14 DAT (Fig. 1a). Those fruits that remained were dissected at harvest, and all but one of the NC had seeds—indicating that they were most likely missed during flower staging and were pollinated, suggesting that the size data for these treatments are likely skewed. NAA alone and GA_3_ + NAA fruit had atrophied ovules and neither senesced in the first 2 months (Fig. 1a) nor did they grow following treatment. NPA also had no obvious fruit growth; NPA-treated fruit senesced early and at a similar rate as NC. It is unknown whether all the large NPA-treated fruits that were retained past 14 DAT were seeded, but the abscission of all but three seeded fruit by 50 DAT indicates that NPA does not cause parthenocarpic development. GA_3_ alone caused the enlargement of treated fruit; although GA_3_-treated fruit had small ovules, fruit initially grew faster in the first 2 weeks following hormone application and then equalized with pollinated controls, ultimately becoming similarly-sized.

### Seed production: GA_3_-treated apples had no seeds

**Fig. 3:**
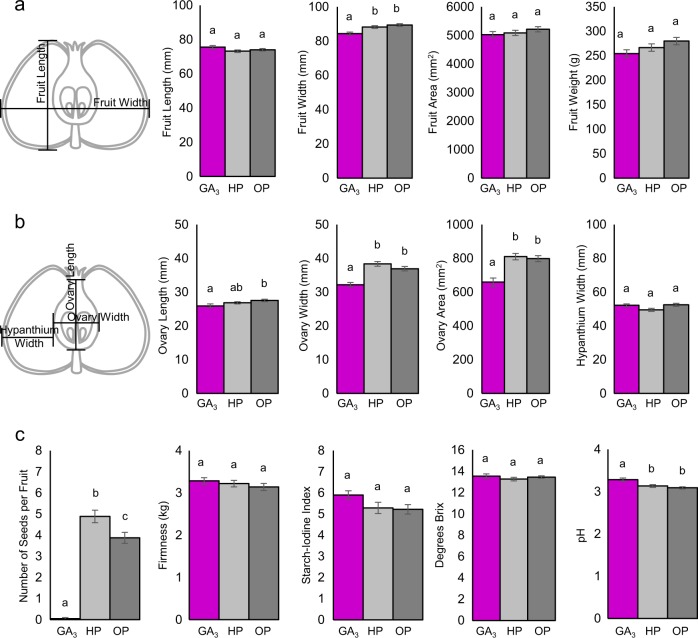
Size, shape, and fruit quality parameters of GA3-treated and pollinated controls at maturity. **a** Diagram illustrating spans used for fruit length and width measurements. Graphs showing average length, width, area, and weight of whole fruits. **b** Diagram illustrating spans used for the hypanthium and ovary width and ovary length. Graphs showing average ovary length, ovary width, ovary area and hypanthium width, calculated by subtracting ovary width from fruit width. **a**, **b** GA_3_-treated (*n* = 50), hand pollinated (*n* = 55), and open pollinated (*n* = 58). **c** Graphs showing average number of seeds at harvest, firmness, starch content, concentration of soluble solids, and acidity. GA_3_-treated (*n* = 39), hand pollinated (*n* = 45), and open pollinated (*n* = 55). Error bars indicate standard error. Letters indicate significant difference determined by ANOVA followed by Tukey HSD

All retained GA_3_-treated and pollinated control apples were collected 132 DAT, when untreated ‘Honeycrisp’ trees in the same block were determined to be at the appropriate stage for commercial harvest. Fruits were dissected laterally, to count the total number of seeds (Fig. 2p, r). The average number of seeds in GA_3_-treated fruit was statistically lower than either pollinated control, with 97% of all GA_3_ fruit being completely seedless (Fig. 3c). HP control fruits had statistically more seeds than OP, illustrating a greater efficiency of hand pollination than insect pollination (Fig. 3c). As stated above, only one other seedless fruit was obtained, from the NC.

### Quality parameters: seedless gibberellin-induced fruits were similar in size and weight, though narrower than pollinated controls at maturity

At maturity, all retained apples were measured, weighed, and then dissected longitudinally to measure the size of their ovaries (cores) (Fig. 2o, q and 3a, b). Average fruit length was statistically similar between GA_3_-treated and pollinated controls (Fig. 3a), but the width of GA_3_-treated fruit was statistically thinner. Medial fruit area and fruit weight were both similar between GA_3_-treated and controls. The average ovary length of GA_3_-treated fruits was similar to HP, but shorter than OP controls (Fig. 3b). The width of GA_3_-treated ovaries was thinner than either pollinated control, and the total ovary area for GA_3_-treated fruit was smaller. Hypanthium width, which was calculated by subtracting ovary width from fruit width, was statistically similar between GA_3_-treated and pollinated controls.

### Gibberellin-induced fruit had similar fruit quality traits at harvest as hand-pollinated controls, though they were less acidic

To determine the effect of hormone applications on fruit quality at harvest, we tested firmness, starch content, sugar content, and acidity. GA_3_-treated fruits were statistically similar to pollinated controls in firmness, starch content, and sugar content, but were statistically less acidic than pollinated controls (Fig. 3c).

### Gene expression patterns: similar patterns were detected between GA_3_-treated and hand-pollinated fruit tissues

Phenotypically, the GA_3_-treated apples were overall very similar to the HP apples, implying that the exogenous GA_3_ induced, mimicked or bypassed the molecular pathways that regulate fruit development such that nearly normal development proceeded. To further understand this process, gene expression profiling utilizing RNA-seq was performed on hypanthium, ovary walls, and seeds/ovules that had been treated with GA_3_, NAA, as well as the NC and HP controls. It was expected that genes with similar expression levels in the GA_3_ and the HP control but different in the NAA and NC would be those genes that were associated with fruit development. Those genes with similar expression in the GA_3_, NAA, and HP control but different in the NC would be associated with fruit retention. Those specific only to the HP control would be associated with either the phenotypic differences affecting shape or acidity as well as the development of the seed. And those specific to the NC would be those genes specific to the senescence and early fruit drop. By 18 DAT, NC fruits exhibited signs of dehiscence. For this reason, RNA-seq was performed on all treatments at this stage, as well as at maturity where applicable.

**Fig. 4:**
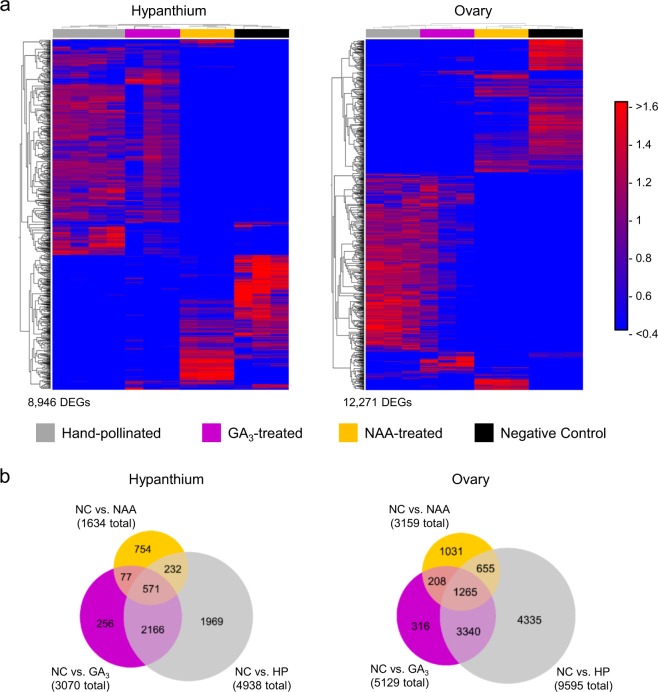
Treatment and tissue dependent patterns of differentially expressed genes (DEGs). **a** Heatmaps showing clustered expression patterns of DEGs by treatment in the hypanthium and ovary at 18 days after treatment. DEGs were identified by comparing across GA_3_-treated, NAA-treated, hand pollinated, and negative control (ANOVA-like), with a fold difference ≥ 2 and a Bonferroni correction value ≤ 0.05. Each treatment contains at least three biological replicates, which cluster by treatment based on similarity in expression pattern. Gray = hand-pollinated, Fuchsia = GA_3_-treated, Yellow = NAA-treated, Black = negative control. Normalized expression values range from < 0.4 (bright blue) to > 1.6 (bright red). Total number of DEGs identified for 18 DAT: hypanthium = 8946, ovary = 12,271. **b** Venn diagrams showing relationships between sets of DEGs. Hand pollinated, GA_3_-treated and NAA-treated are compared with negative control. DEGs are determined with a fold difference ≥ 2 and a Bonferroni correction value ≤ 0.05. HP hand pollinated, GA_3_ GA_3_-treated, NAA NAA-treated, NC negative control

In order to analyze overall RNA expression patterns, we constructed heatmaps comparing differently expressed genes (DEGs) from multiple treatments in each tissue type at 18 and 132 DAT. Comparing all four treatments at 18 DAT and isolating DEGs with a fold difference of ≥ 2 and a Bonferroni corrected level of significance of ≤ 0.05, led to 8946 DEGs in the hypanthium and 12,271 in the ovary (Fig. 4a). Clustering samples statistically led to individual replicates clustering by treatment, with more similarity in expression patterns between HP control and GA_3_-treated tissues, compared with NAA-treated or NC.

To analyze RNA expression at 18 DAT in a complimentary way, we determined the DEGs relative to the NC, leading to a total of 4938 DEGs in the HP hypanthium, 3070 in GA_3_-treated, and 1634 in NAA-treated (Fig. 4b). When the relationships between these sets of DEGs were examined to see which were in common using a Venn diagram, the largest quantity (2166) fell into the category shared between HP and GA_3_-treated. Each group was then analyzed using MapMan to determine if there was an enrichment in any functional category of genes. The shared HP and GA_3_-treated category, and the NAA-treated unique category were the only groups with statistically enriched bins (Figure S2a). Bins in the shared category between HP and GA_3_-treated consisted of pathways such as cell wall degradation, cell wall modification, lipid metabolism, secondary metabolism, regulation of transcription (WRKY transcription factor family), and signaling receptor kinases. In the category unique to NAA treated, enriched bins consisted of stress, regulation of transcription, and signaling pathways. No other categories contained enriched bins. In the ovary, there were more DEGs in each comparison; compared with NC, 9595 genes were differentially expressed in the HP ovary, 5129 in GA_3_-treated, and 3159 in NAA-treated (Fig. 4b). The largest quantity of DEGs (4335) fell into the category specific to HP. This category contained the statistically enriched bins: cell wall degradation, lipid metabolism, hormone metabolism, regulation of transcription (WRKY transcription factor family), protein, protein degradation, signaling receptor kinase, cell, cell organization, and cell cycle (Figure S2b). As in the hypanthium, the category shared between GA_3_-treated and HP also contained enriched bins, including cell wall, lipid metabolism, secondary metabolism, stress, redox, regulation of transcription (WRKY transcription factor family, protein, protein degradation, signaling receptor kinases, cell, cell organization, and cell cycle. The category specific to NAA-treated also contained enriched bins consisting of stress and signaling pathways. No other categories contained enriched bins.

**Fig. 5:**
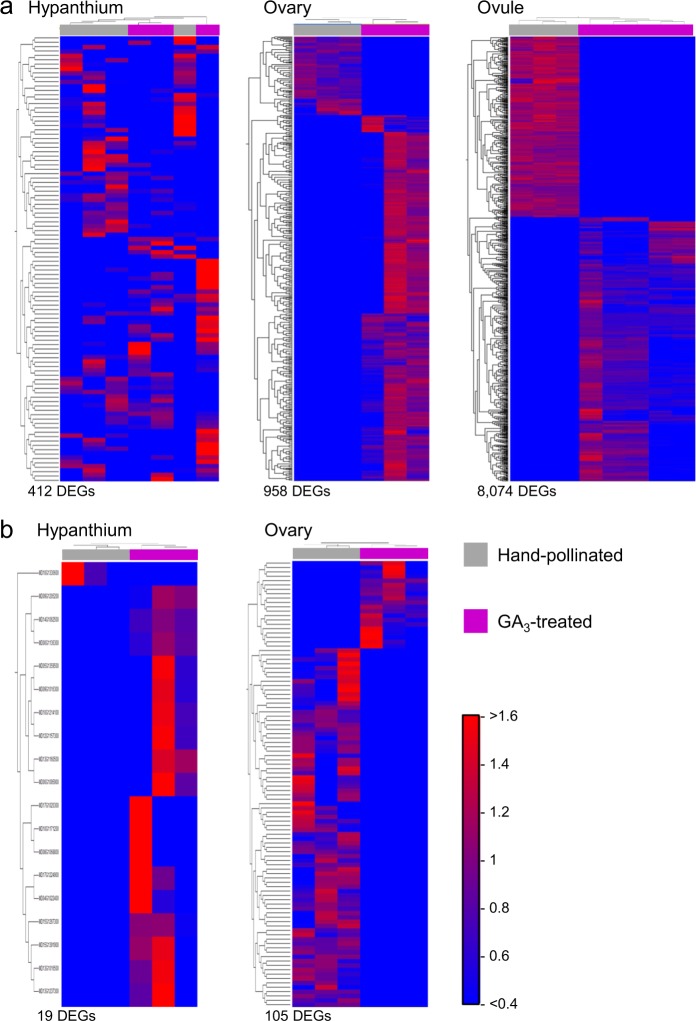
Heatmaps showing clustered expression patterns of differentially expressed genes (DEGs) from a GA_3_-treated and hand-pollinated hypanthium, ovary, and ovule. **a** 18 days after treatment (DAT). **b** 132 DAT. DEGs were identified by comparing GA_3_-treated and hand pollinated with a fold difference ≥ 2 and a Bonferroni correction value ≤ 0.05. Each treatment contains at least three biological replicates which cluster based on similarity in expression pattern. Gray = hand-pollinated, Fuchsia = GA_3_-treated. Normalized expression values range from < 0.4 (bright blue) to > 1.6 (bright red). Total number of DEGs identified for 18 DAT: hypanthium = 412, ovary = 958, ovule = 8074, 132 DAT: hypanthium = 19, ovary = 105

We also compared GA_3_-treated directly to HP tissues at 18 DAT and at maturity (Fig. 5). At 18 DAT, there were 412 DEGs in the hypanthium, 958 DEGs in the ovary, and 8074 in the ovule (Fig. 5a). Hypanthium samples for this time point did not completely cluster by treatment. When DEGs from each tissue type were separated into up- and downregulated categories and analyzed using MapMan, we identified a number of enriched bins (*p* ≤ 0.05) (Table S3). DEGs upregulated in HP vs. GA_3_-treated yielded five bins for the hypanthium, seven for the ovary, and 122 for the ovule, while those downregulated in HP vs. GA_3_-treated yielded four bins for the hypanthium, 20 for the ovary, and 125 for the ovule. Enriched bins were identified using an uncorrected *p*-value, as more stringent selection yielded no results for the hypanthium or ovary samples. In the upregulated HP hypanthium category, the protein bin was enriched; in the downregulated HP hypanthium category, enriched bins included lipid metabolism, hormone metabolism (ethylene synthesis/degradation), and protein. In the ovary, the upregulated HP category-enriched bins included cells (cell division and cell organization) and fermentation; the downregulated HP ovary category-enriched bins included lipid metabolism, secondary metabolism, hormone metabolism (cytokinin synthesis/degradation), protein, development, and transport. In the ovule, there were many enriched bins, corresponding to the higher number of both up- and downregulated DEGs we analyzed and the lower stringency we used. Many of the major pathways were enriched in both up- and downregulated DEG lists. Some notable differences included PS, major CHO, minor CHO, glycolysis, fermentation, TCA, mitochondrial electron transport, and nucleotide metabolism pathways, which were only enriched in the upregulated HP ovule, and co-factor and vitamin metabolism, redox, biodegradation of xenobiotics, and signaling, which were only enriched in the downregulated HP ovule. At maturity (132 DAT), comparing GA_3_-treated to HP resulted in 19 DEGs in the hypanthium and 105 in the ovary (Fig. 5b). Only one gene was upregulated in the HP hypanthium, while downregulated HP hypanthium DEGs were enriched for stress and cell wall bins. In the ovary, there were no significant enriched bins in the upregulated DEGs, while hormone gibberellin synthesis–degradation was enriched in the downregulated HP ovary category (Table S3).

### Comparison of cell size in GA_3_-treated and hand-pollinated ovary and the hypanthium

To determine whether the difference in ovary width was due to cell division or cell expansion, we sectioned the hypanthium and ovary tissues and counted and measured cells (Figure S3). Cell size was similar between GA_3_-treated and the HP hypanthium, however, we found significantly smaller cells in the GA_3_-treated ovary (*p* ≤ 0.001, Figure S3b). Numbers of cells per section negatively correlated with cell size, as more cells were visible to count when they were smaller. Similar numbers of cells in hypanthium samples support the similarity in cell size between treatments (Figure S3c). Given the observed difference in ovary cell size, we identified several genes within the cell wall expansion and cell wall modification pathways that are differentially expressed between GA_3_-treated and HP (Table S4). These genes encompass a number of families, including expansins, xyloglucan endotransglycosylases, and pectin esterases.

### Candidate genes affecting fruit quality traits

**Fig. 6:**
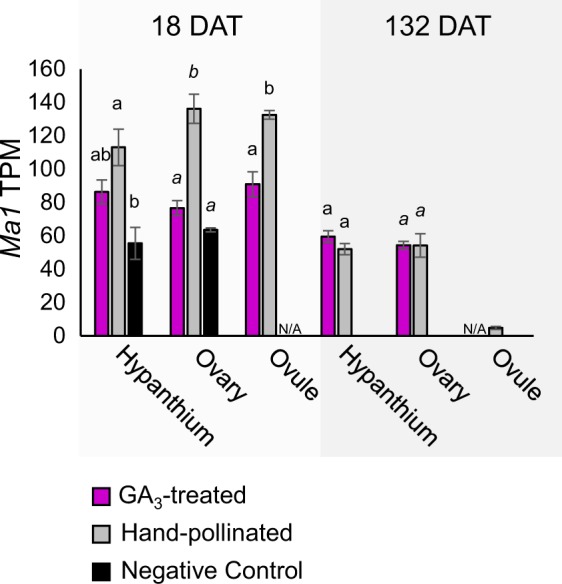
Expression of the apple acidity gene *Ma1*. *Ma1* expression is significantly lower in GA_3_-treated ovary and ovule compared to hand pollinated at 18 days after treatment (DAT). Identical letters indicates statistical similarity (*p* ≤ 0.05); significance was calculated within tissue type using ANOVA and Tukey HSD. Error bars indicate standard error. TPM = transcripts per million

Parthenocarpic GA_3_-treated fruit were similar in quality to HP fruit at harvest, although they were less acidic and had narrower ovaries. To compliment the DEG analyses, we performed to investigate the genetic causes of these morphological differences, we also examined a number of candidate genes known to regulate either fruit acidity or fruit shape. For acidity, we focused on the malate-transporter *Ma1*, which has been shown to correlate with acidity in apple^25^. At 18 DAT, the *Ma1* gene (MDP0000252114) was expressed significantly lower in GA_3_-treated ovary and ovule tissues, compared with HP (Fig. 6). By maturity, there were no differences.

To further explore the control of fruit shape, we analyzed expression of genes known to control fruit shape and size in tomato. Both *SUN* and *OVATE* are genes that have been determined to be involved in fruit length-to-width ratios in tomato, while *CNR* and *KLUH* are genes that have been shown to affect tomato fruit size^26^. We identified the two most-likely orthologs for each of these genes in the apple genome based on sequence similarity and analyzed their expression using our transcriptome data (Figure S4). While the *SUN* ortholog MD02G1297300 showed significantly less expression in GA_3_-treated ovule compared with HP at 18 DAT, the other ortholog MD14G1079600 showed low expression in all tissues at both time points and was not analyzed statistically for differences in expression. The *OVATE* ortholog MD02G1151100 did not show any significant differences between GA_3_-treated and HP tissues, while MD15G1030700 exhibited low expression. *CNR* orthologs MD10G1094000 and MD05G1079900 both show low expression overall. *KLUH* ortholog MD13G1060500 was expressed in HP ovule at 132 DAT, but GA_3_-treated ovules were atrophied at this time and so comparison was unfeasible. The other *KLUH* ortholog MD16G1059700 was expressed at low levels.

## Discussion

The primary goals of this work were to determine if exogenous hormone applications could stimulate parthenocarpic ‘Honeycrisp’ apple development, to determine whether parthenocarpic fruit had quality differences at maturity, to provide a catalog of genes and pathways affected/effected by parthenocarpy, and to investigate genetic causes of any observed morphological differences.

In our study, we examined the effect of two hormones, gibberellic acid (GA_3_) and synthetic auxin (NAA), as well as an auxin-transport inhibitor (NPA). Given that the combined effect of auxin and GA has been shown to be more effective at increasing retention in other species and other apple cultivars, we expected the same to be true in ‘Honeycrisp’. However, the NAA alone or in combination with GA_3_ stimulated retention but arrested development. This could have been the result of using a high concentration of NAA, suggesting that excess auxin interferes with the senescence process responsible for un-pollinated fruit drop. The effect of exogenous hormone application on fruit size and shape has been shown to be variable in different species with different fruit types. In *Arabidopsis*, a dry fruit, neither single nor combined treatments with synthetic auxins (2,4-D or NAA) and GA_3_ caused parthenocarpic fruits to enlarge to a comparable size as pollinated controls^6^. In strawberry, another accessory fruit with fleshy receptacle tissue and dry achenes, the combined effect of NAA and GA_3_ is needed in order to cause the receptacle to enlarge to a similar size as pollinated controls^11^. In apple, the size effects of GA alone or in combination with auxin or cytokinin are variable (literature results summarized in Table S5). Treated fruits have been reported as larger, smaller, or similar in size, though a common feature is an increase in the length-to-width ratio. We observed a similar effect in the shape of GA_3_-induced ‘Honeycrisp’ fruit compared with pollinated controls, which we determined was caused by a difference in the ovary width, independent of hypanthium width. This indicates that although GA_3_ was sufficient to stimulate normal hypanthium development, it was unable to trigger a normally developing ovary. There is some evidence to suggest that fruits may have been wider if exogenous cytokinin had been included in the GA_3_ treatment, as cytokinin applications have been shown to increase the size of apple and pear fruit without increasing the L/D ratio^20,27,28^. Unfortunately, it is unknown whether this effect was due to the expansion of the ovary, hypanthium, or both.

Cytokinin is known to be involved in cell division, and an interesting outcome of the analysis of HP vs. GA_3_ DEGs at 18 DAT was that the cytokinin synthesis/degradation pathway was enriched in the DEGs that were downregulated in HP compared with GA_3_-treated ovary (Table S3). These genes consist of six cytokinin oxidase/dehydrogenase and UDP-glucosyl transferases, which are putatively involved in the degradation or sequestration of cytokinins. This supports the theory that a lack of cytokinin is responsible for the thinner ovary of GA_3_-treated apples, and suggests that while GA_3_ may induce a cascade of signals that lead to a mature fruit, there are divergent signals in GA_3_-treated ovaries that limit the levels of bioactive cytokinin, causing less cell division. Whether controlled by cytokinin or other signals, genes involved more directly in cell division also differ between HP and GA_3_-treated ovary. In DEGs that are upregulated in HP ovary, the cell division pathway is enriched with four genes (Table S3, S4); one involved in the G2/M transition and three related to the metaphase/anaphase transition. The G2/M transition gene is *MdCDKB2;1*, and has been shown to be positively associated with cell division in apple^29^.

Because of the enrichment in the cell division pathway in HP compared with GA_3_-treated ovary, it is likely that cell division was responsible for the observed ovary width difference. To verify this, we analyzed cell size, expecting to find similarly sized cells between the two treatments. While we did find statistically similar cells in HP and GA_3_-treated hypanthium, GA_3_-treated ovaries had smaller cells, indicating that it is possible that the ovary size difference is caused by differences in cell expansion as opposed to, or possibly in combination with, cell division. For this reason, we reexamined the genes that were differentially expressed between HP and GA_3_-treated ovary. While the cell wall degradation and cell wall modification pathways are not enriched at either 18 or 132 DAT, DEGs do fall within these pathways and may be involved in cell expansion differences between the two treatments (Table S4). Interestingly, although we observed larger cells in the HP ovary, a larger number of cell wall modification/degradation genes are upregulated in GA_3_-treated ovary at both 18 and 132 DAT. Taken together, the differential expression in both cell division and cell expansion genes make it likely that one or both of these processes are responsible for thinner GA_3_-treated ovaries. While the differences we observed in cell size indicate that cell expansion is a factor, a more complete entire ovary analysis would likely be needed to definitively determine the role of each process, especially given the large range of cell sizes in different regions throughout the ovary.

In order to expand our investigation into the molecular cause of ovary shape differences, we examined the expression of genes known to be involved in fruit weight and shape in tomato, a fruit that is entirely ovary-derived^26^. *SUN* and *OVATE* have been shown to control tomato fruit elongation. *SUN* is active both pre- and post fertilization, and alters cell division; when it is overexpressed, it causes a redistribution of mass from the mediolateral access to the proximal–distal axis resulting in an elongated fruit^30^. In apple, there is lower expression of the *SUN* ortholog MD02G1297300 in GA_3_-treated ovules at 18 DAT, when compared with HP ovules, indicating that this gene is likely not responsible for the increased L/D ratio we observed in GA_3_-treated fruit. *OVATE* functions pre-anthesis and affects ovary shape by regulating growth along the proximal–distal axis at the proximal end of the fruit. We observed no differences in *OVATE* ortholog expression between GA_3_-treated and HP tissues, indicating that this gene is also likely not involved. We also examined the expression of the fruit weight genes *CNR* and *KLUH*. *CNR* ortholog expression was low in apple, which may be expected given the fact that this gene is active pre-anthesis in tomato. One *KLUH* ortholog, MD13G1060500, is expressed in the HP ovule at maturity, which corresponds to expression in tomato. This gene is thought to promote organ growth using non-hormonal signals, and since GA_3_-treated fruit lack MD13G1060500 expression (seedless) and have smaller ovaries, this gene may possibly be involved in stimulating apple ovary growth.

‘Honeycrisp’ is a cultivar released by University of Minnesota in 1990, which is prized by consumers for its particularly large size, sweet taste, and crisp texture^31^. Quality traits are particularly important for ‘Honeycrisp’ fruit. In an economic study aimed at determining the potential impacts on grower profits, even relatively small decreases in size or soluble solids concentration (SSC) in ‘Honeycrisp’ apples resulted in substantial monetary losses^32^. Previous studies have shown that commercial sprays containing GA_4_GA_7_ may speed the rate of maturation in ‘Honeycrisp’, causing softer, less starchy, and less acidic fruit at time of harvest^17^. Similar effects have been seen in cultivars ‘Starkrimson Delicious’, ‘McIntosh’, and ‘Empire’^15,19^. GA has been implicated in speeding up the maturation of *Arabidopsis* as well, as GA_3_ application accelerates the destruction of the inner endocarp ena layer in the *Arabidopsis* fruit^6^, which normally does not occur until later developmental stages^33^. GA_3_ has also been shown to accelerate the ripening of apricot when introduced pre-anthesis^34^. To test whether GA_3_ caused a premature ripening in ‘Honeycrisp’, we compared firmness, starch content, sugar content, and pH of GA_3_-treated fruit to pollinated controls at maturity. We did not observe a statistically significant difference in firmness or starch conversion between GA_3_-treated and positive controls; however, pH was statistically higher in GA_3_-treated fruit. The acceptable range of fruit acidity of dessert (table) apples is pH 3.1–3.8, with fruits below this range considered too sour and those higher tasting flat or flavorless^35^. With an average pH of 3.28, the GA_3_-treated apples fell well within this range.

*Ma1* is an aluminum-activated malate transporter (AAMT)-like gene, which has been identified as a major factor in determining apple acidity levels, with higher expression correlating with more acidic cultivars^25^. For this reason, we examined the expression of *Ma1* in HP, NC, and GA_3_-treated hypanthium, ovary, and ovule tissues. The lower expression of *Ma1* in GA_3_-treated ovary and ovule correlates to the higher pH we observed in this treatment; although pH was measured in juice extracted from the hypanthium, which does not show a significant difference in expression. By 132 DAT, the differences in *Ma1* expression were no longer observed, indicating that pH difference is not necessarily an effect of accelerated ripening and instead may be determined much earlier in development. This is consistent with the accumulation patterns of malic acid, which peak early in development and then taper as the apple fruit matures^36^. Interestingly, the levels of *Ma1* expression in NC hypanthium and ovary at 18 DAT were statistically similar to the low GA_3_-treated levels, indicating that *Ma1* regulation may be dependent on some pollination- or fertilization-specific factor, which is not supplied by ectopic GA_3_ application. Alternatively, GA_3_ may downregulate *Ma1*, either directly or indirectly. It is important to note that while we observed a difference in pH, additional experiments would be needed to verify whether the lower levels of acid in GA_3_-treated fruit are in fact due to lower levels of malic acid, as opposed to one of the other, less abundant organic acids. Likewise, additional experiments would be needed in order to determine any relationship between GA and *Ma1* expression.

## Conclusion

Although exogenous hormone applications have the potential to improve apple yield and quality, little is known about the developmental role hormones play in fleshy accessory fruits. In this study, we showed that exogenous GA_3_ is sufficient to either induce, mimic, or bypass the typically fertilization-dependent molecular pathways that stimulate fruit development, resulting in parthenocarpic seedless fruit. By analyzing fruit morphology in detail, we showed that the resulting shape differences in these parthenocarpic fruit are due to thinner ovaries, and we identified a number of genes that are likely involved in this difference. We also showed that GA_3_-induced fruit are less acidic, and we propose a likely gene candidate for this difference as well. Additionally, we identified several genetic pathways that are likely involved in processes like fruit set and fruit flesh development, which should help with future investigations in apple and in other species.

## Materials and methods

### Exogenous hormone treatments

Nine-year-old ‘Honeycrisp’ apple trees on EMLA 7 rootstock in Kearneysville, WV were used for hormone studies. The block was located on Hagerstown silt loam soil and was not irrigated. Trees were spaced 12’ × 16’, trained to a central leader system and maintained using a hormone-free commercial spray program. Trees were treated in April 2017 with spray applications of 2.9 mM gibberellic acid (GA_3_) (Sigma Chemical Company, MO), 2.9 mM 1-naphthaaleneacetic acid (NAA) (Sigma Chemical Company, MO), 100 µM 1-n-naphthylphthalamic acid (NPA) (Chem Service, PA), and 2.9 mM GA_3_ + 2.9 mM NAA. GA_3_ was initially dissolved in 1 ml of 95% ethyl alcohol (Warner-Graham, MD), NAA in 1 ml of 5 M potassium hydroxide (Sigma Chemical Company, MO), and NPA in 2 ml of DMSO (Fisher Scientific, NJ). Solutions were diluted to 1 L with deionized water and 0.2 ml of Tween20 surfactant (Bio-Rad, CA) was added. A 1 L solution containing 1 ml of 95% ETOH, 5 M KOH, 100% DMSO, and 0.2 ml of 100% Tween20 was also applied as a negative control. One tree was used for each treatment and was divided into three equal replicates. Both tightly closed and open flowers were removed, and the number of remaining balloon stage flowers was recorded. Solutions were applied by spraying flowers until run-off, and all trees except for open-pollinated treatments were then covered with 50% white shade cloth (Green-tek, CA) for 9 days to exclude pollinators while stigmas were receptive. Each tree received one of the seven treatments consisting of GA_3_, NAA, NPA, GA_3_ + NAA, negative control spray, unsprayed and hand pollinated, and unsprayed and open pollinated.

### Quantifying retention and growth of developing fruit

Fruits were counted and measured with digital calipers (Mitutoyo, IL) every 2 weeks over a 2-month period to determine treatment effects on retention and size. The longest and widest dimension of each fruit was used to determine size by calculating the area of an ellipse (Area = π x radius 1 × radius 2). Fruits were collected at various stages and photographed with a Nikon d5200 DSLR (Nikon, Japan).

### Determining quality of mature fruit

All remaining fruits were harvested at maturity, (132 DAT), separated by treatment and reps and evaluated for fruit and ovary size, total weight, maturity, and ripeness. The longest and widest axis of each halved fruit and of the respective ovary was measured. Hypanthium width was calculated by subtracting the width of the ovary from the total fruit width. Weight was measured on an electronic balance, and internal fruit firmness was measured using a FT 327 penetrometer (Wagner, CT) mounted on a stand. Starch index rating was determined following the iodine-staining protocol detailed by the Cornell Cooperative Extension^37^. Fruit juice pH was measured with 0.0–6.0 pH test strips (Sigma-Aldrich, MO), and degrees Brix was determined with a Pocket Pal-1 digital refractometer (Atago, WA).

### RNA-sequencing

Fruits from HP, NC, GA_3_-treated, and NAA-treated trees were collected at 18 days after treatment (DAT), and mature fruits from HP and GA_3_-treated trees were collected at 132 DAT. Three biological replicates from each treatment and date were collected, containing tissue from one to three fruit. The hypanthium, ovary, and ovule/seed tissues were immediately separated by dissection, frozen in liquid nitrogen, and lyophilized. RNA was isolated from lyophilized tissue using Norgen Plant/Fungi Total RNA Purification Kit (Norgen Biotek Corp., ON) following the manufacturer’s protocol. DNA was removed from RNA using TURBO DNA-free kit (Thermo Fisher Scientific, MA) according to the manufacturer’s protocol. DNased RNA quality and purity was assessed by electrophoresis on a 1% agarose gel and visualized on a Typhoon FLA 9500 scanner (GE Healthcare Life Sciences, IL), and by analyzing spectrophotometrically on a NanoDrop^®^ ND-1000 (Thermo Fisher Scientific, MA). RNA was sent to GENEWIZ (GENEWIZ Inc., NJ) where 45 RNA libraries were constructed using NEB Next^®^ Ultra™ RNA Library Prep Kit for Illumina^®^ with polyA selection (New England Biolabs Inc., MA) with total RNA and were sequenced with an Illumina Hiseq 2000 (Illumina Inc., CA). Approximately 30–50 million paired-end 150 bp reads were obtained per sample and analyzed with CLC Genomics v.11 (Qiagen, MD). One HP hypanthium sample and two GA_3_-treated ovule samples at 18 DAT were re-sequenced and these were also included in all gene expression analyses.

### Analysis of gene expression patterns in apple tissues

RNA sequences were filtered for quality scores using defaults from CLC Genomics Workbench v.11 and then sequences matching ribosomal (*P. persica*), mitochondrial (*M. domestica*), and chloroplast (*M. domestica*) sequences were filtered out using the Map Reads to Reference function. The remaining sequences were mapped using the RNA-seq analysis function to the annotated *Malus x domestica* GDDH13 Whole Genome v1.1^38^. Differentially expressed gene (DEG) lists were generated using the differential expression for RNA-seq function (fold difference ≥ 2, Holm Bonferroni ≤ 0.05). These were used to filter by statistics when creating heatmaps, which were generated using the create heatmaps for RNA-seq function. Venn Diagrams were generated using the create Venn diagram for RNA-seq function (fold difference ≥ 2, Holm Bonferroni ≤ 0.05). Gene lists generated for the Venn diagrams were analyzed in MapMan v. 3.6^39^ for enriched bins using Wilcoxon rank sum test with Benjamin Hochberg correction. MapMan bins for apple proteins were assigned using Mercator Pipeline for automated sequence annotation^40^.

### Comparison of cell number and size

Three replicate ovary and three replicate hypanthium tissue samples were dissected from mature (132 DAT) GA_3_-treated and HP fruit and embedded for sectioning^41^. Briefly, tissue was fixed in 4% paraformaldehyde (Affymetrix USB, OH) for 1e week, dehydrated through an ethanol series, and embedded in Paraplast Plus paraffin wax (Sigma-Aldrich, MO). Embedded tissue was sectioned at 8 µm with an 820 rotary microtome (American Optical, NY) and mounted on Superfrost plus microscope slides (Thermo Fisher Scientific, MA). Sections were washed with Histo-Clear II (National Diagnostics, GA), rehydrated and stained with 0.05% Toluidine Blue (Electron Microscopy Sciences, PA). Stained slides were viewed at 10x on an Optiphot-2 light microscope (Nikon, JPN); digital images containing ~1.3 × 1 mm spans of the largest observed cells were taken with a PAXcam (MIS Inc, IL). Three images from each replicate were analyzed by counting and measuring cells using ImageJ^42^. Cell size was averaged for nine images per tissue type for each treatment and compared statistically between treatments using an unpaired-*t* test.

### qPCR and validation of RNA-seq data

Quantitative real-time PCR on total RNA was conducted with an ABI PRISM^®^ 7900 Sequence Detection System (Thermo Fisher Scientific, MA) on five genes, to validate transcript per million (TPM) values from the transcriptome. Amplifications were completed with Invitrogen Superscript III Platinum SYBR Green One-Step qPCR kit with ROX (Thermo Fisher Scientific, MA). Primers (Figure S1) were validated prior to use, to confirm single product amplification and consistent efficiencies through dilution curves. RNA samples were run in triplicate and normalized to Apple *TRANSLATION ELOGATION FACTOR 2* (*MdTEF2*) using the 2^∆∆^Ct method^43^. All values were standardized to GA_3_-treated hypanthium and compared with both identically-standardized TPM values from RNA-seq, and TPM values that had first been standardized to *MdTEF2* (Figure S5).

## Supplementary information

Figure S3

Figure S4

Table S2

Figure S1

Figure S2

Figure S3

Figure S4

Figure S5

Table S1

Table S2

Table S3

Table S4

Table S5

Supplementary File

## Data Availability

RNA-sequencing data is available through Sequence Read Archive (SRA), accession number PRJNA521965.
