# Supplementary figures and images for: Gibberellic acid induced parthenocarpic ‘Honeycrisp’ apples (Malus domestica) exhibit reduced ovary width and lower acidity
Source: Hortic Res. 2019 Apr 6;6:41. doi: 10.1038/s41438-019-0124-8 (PMC6441655; doi:10.1038/s41438-019-0124-8)

Figure S3

a

## Ovary Wall

## Hypanthium

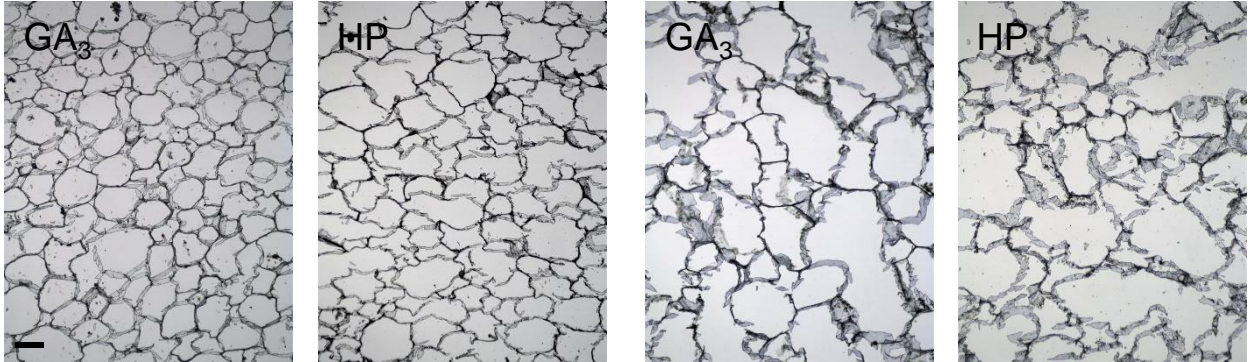

**b**

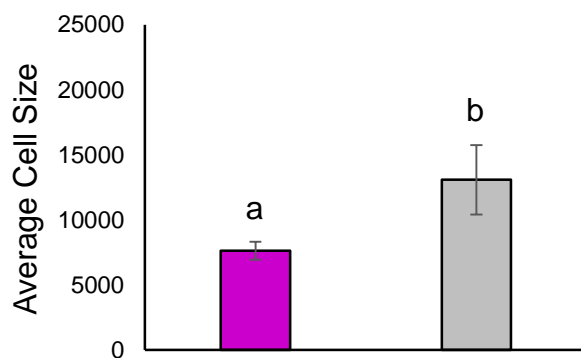

C

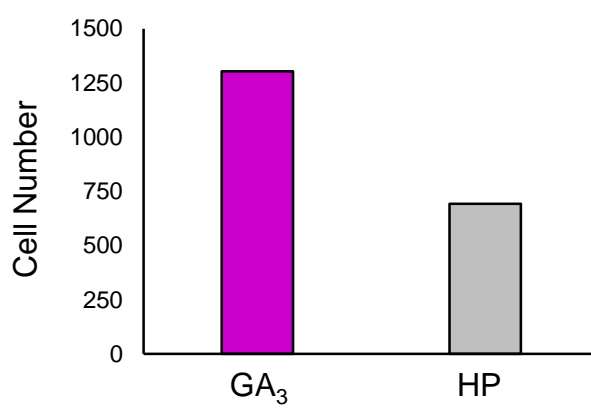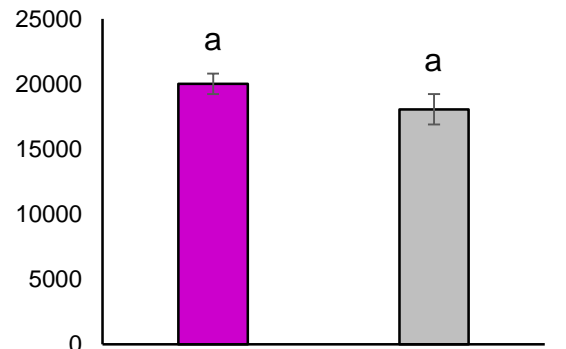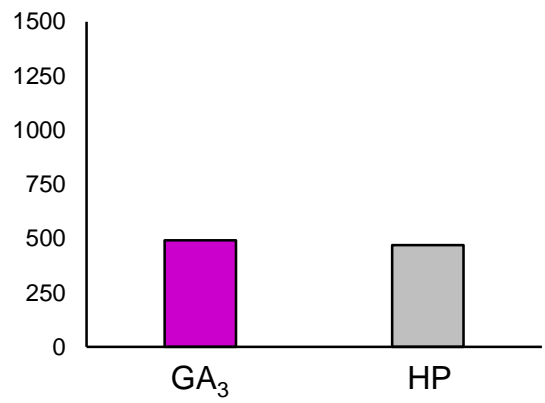

Supplement: Supplementary file 1 — Figure S3 [file 41438_2019_124_MOESM1_ESM.pdf]

Figure S4

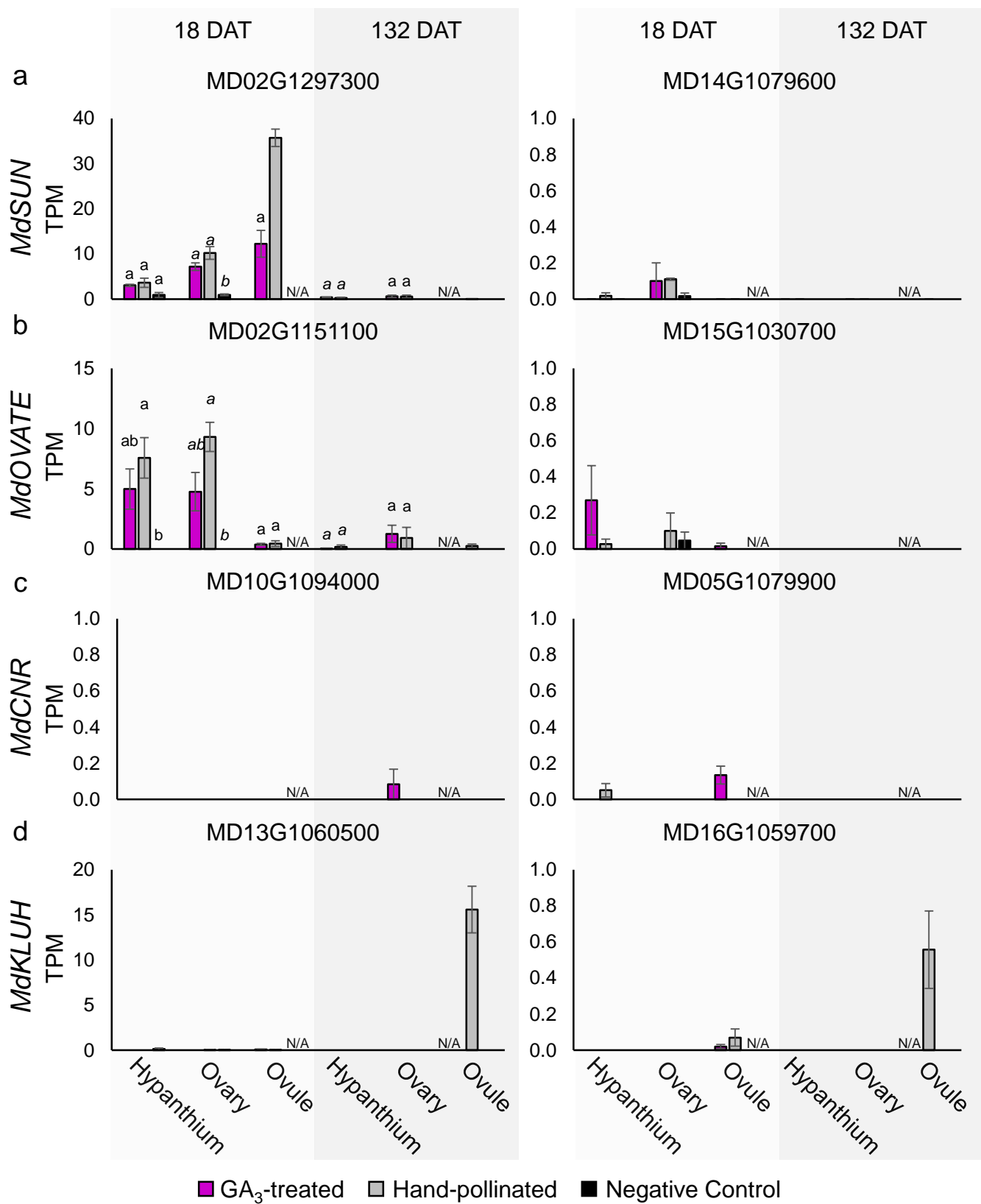

Supplement: Supplementary file 2 — Figure S4 [file 41438_2019_124_MOESM2_ESM.pdf]

Figure S1

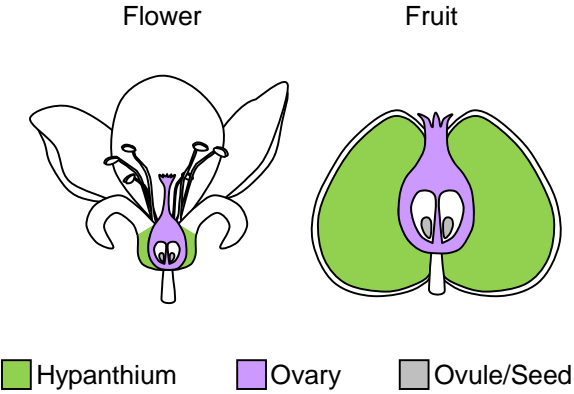

Supplement: Supplementary file 4 — Figure S1 [file 41438_2019_124_MOESM4_ESM.pdf]

Figure S2

A. Hypanthium

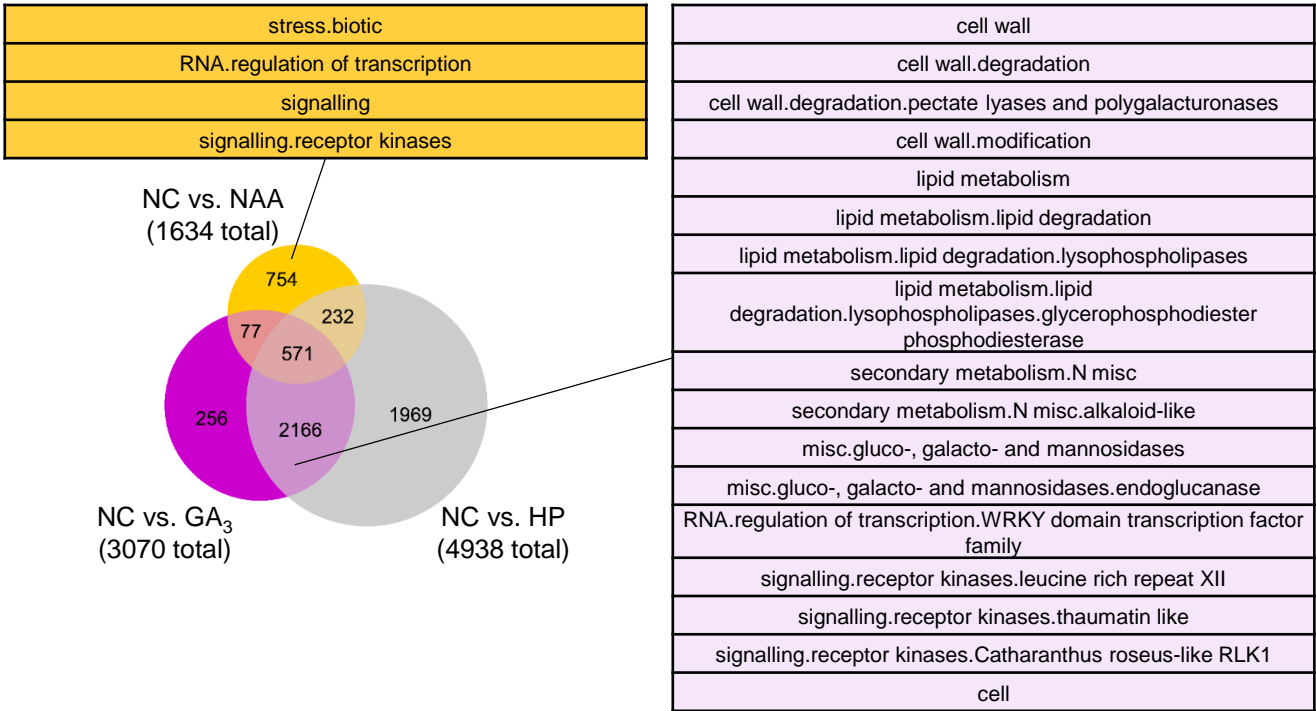

B. Ovary

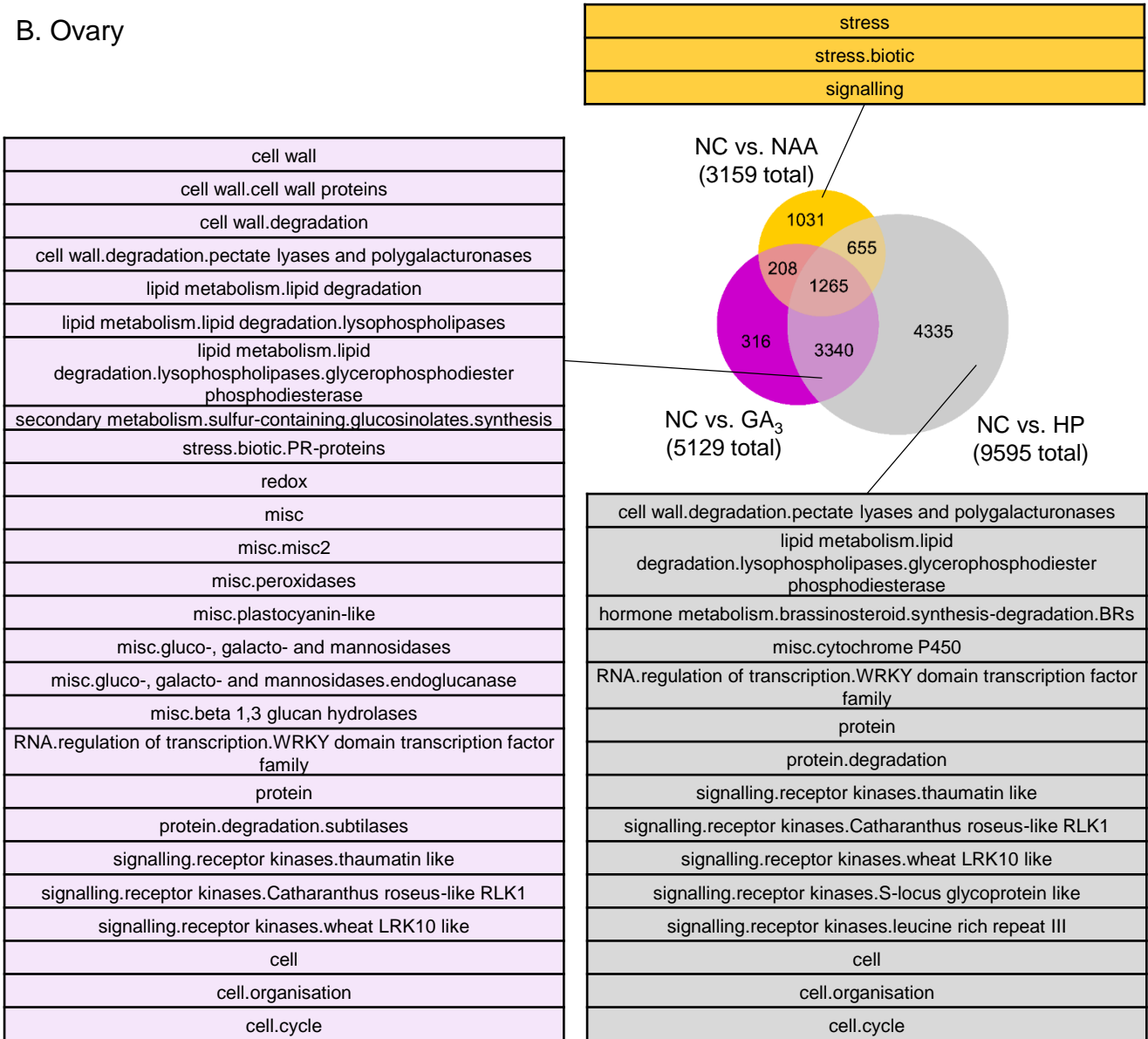

Supplement: Supplementary file 5 — Figure S2 [file 41438_2019_124_MOESM5_ESM.pdf]

Figure S3

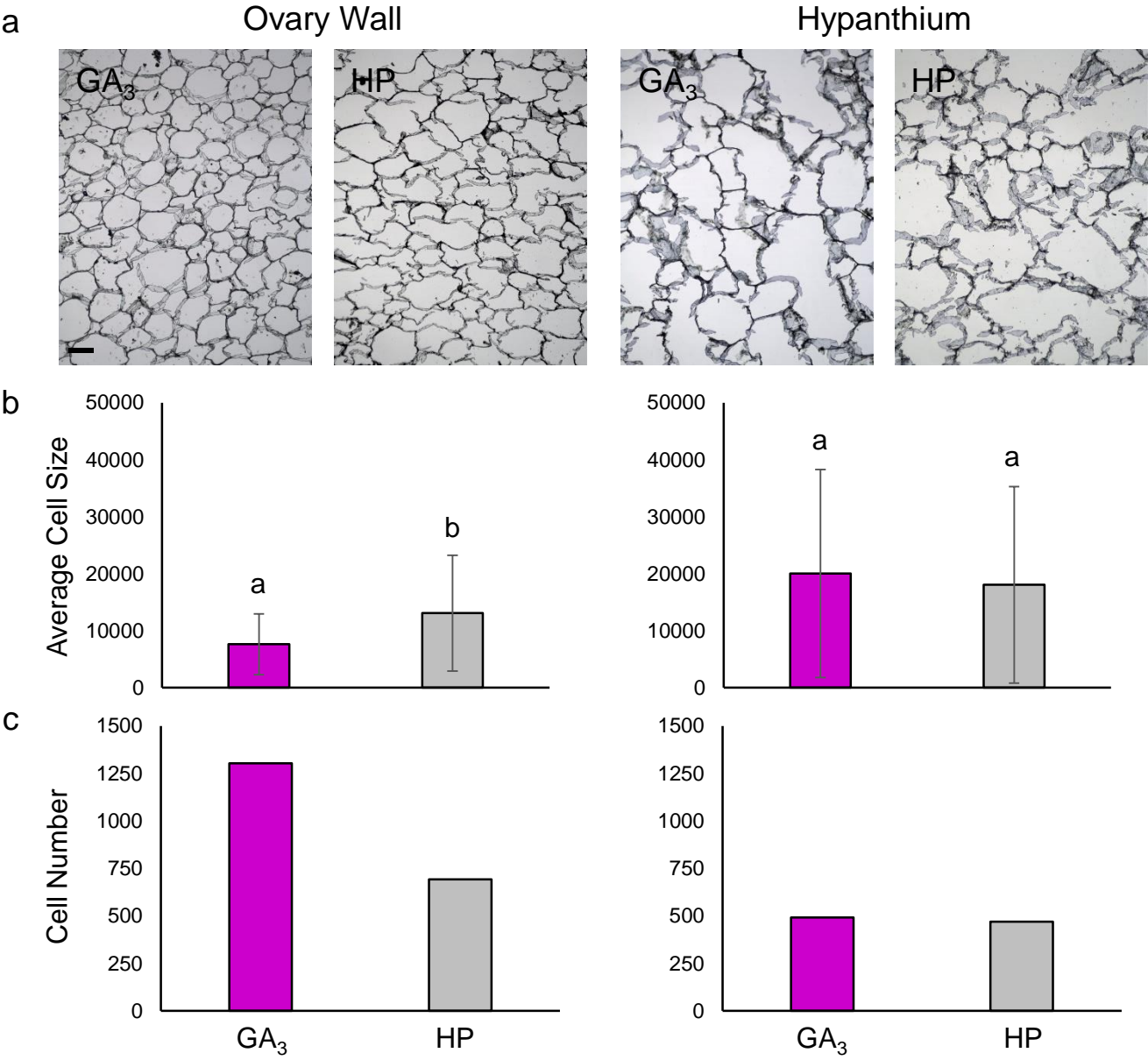

Supplement: Supplementary file 6 — Figure S3 [file 41438_2019_124_MOESM6_ESM.pdf]

Figure S4

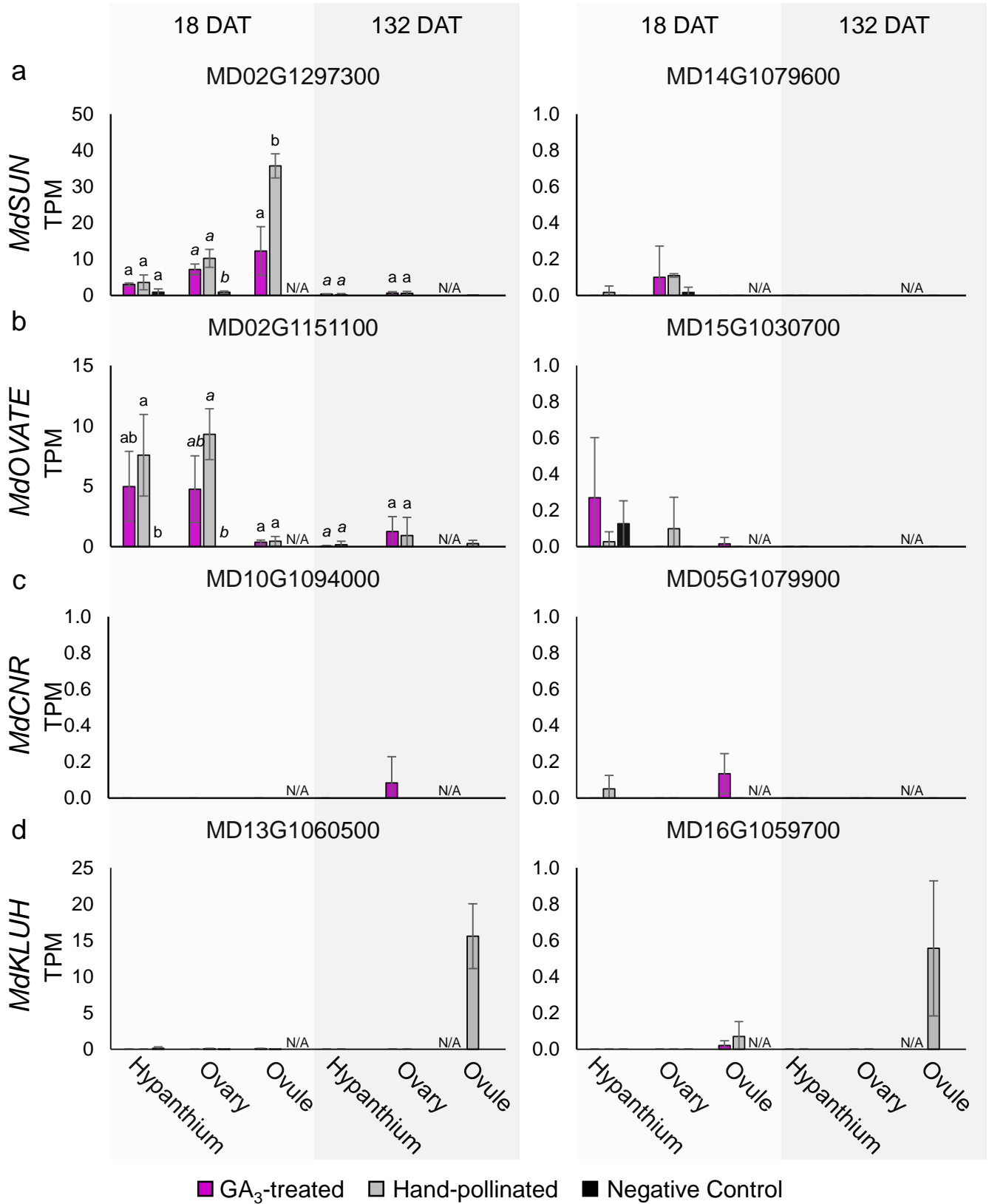

Supplement: Supplementary file 7 — Figure S4 [file 41438_2019_124_MOESM7_ESM.pdf]

Figure S5

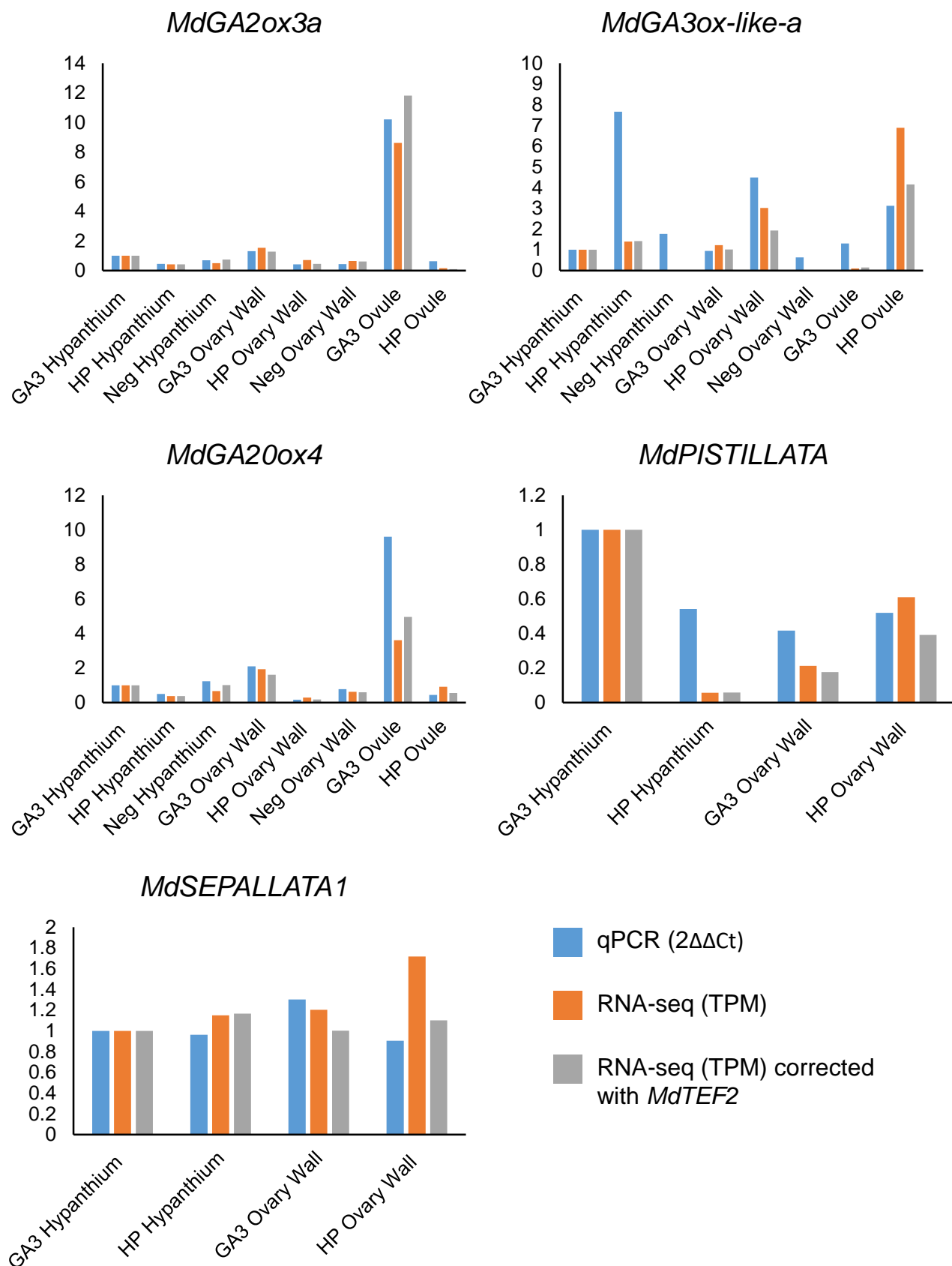

Supplement: Supplementary file 8 — Figure S5 [file 41438_2019_124_MOESM8_ESM.pdf]
