# Supplementary material for: Gibberellic acid induced parthenocarpic ‘Honeycrisp’ apples (Malus domestica) exhibit reduced ovary width and lower acidity
Source: Hortic Res. 2019 Apr 6;6:41. doi: 10.1038/s41438-019-0124-8 (PMC6441655; doi:10.1038/s41438-019-0124-8)
Supplement: Supplementary file 3 — Table S2 [file 41438_2019_124_MOESM3_ESM.pdf]

Table S2

Average size of fruit					
Treatment	14 DAT	28 DAT	36 DAT	50 DAT	131 DAT
GA_3	56.96 A	323.24 A	869.61 A	1606.34 AD	5026.61 A
NAA	21.84 B	27.75 B	33.80 B	20.25 B	NA
GA_3 + NAA	26.23 B	34.47 B	31.55 B	53.12 B	NA
NPA	23.78 B	322.19* A	542.57* C	673.48* C	NA
Negative Control	21.23 B	279.99* A	354.94* C	1340.38* A	NA
Hand Pollinated	43.03 C	445.10 A	963.35 A	1684.24 D	5086.33 A
Open Pollinated	42.72 C	445.37 A	1100.19 D	1765.12 D	5213.57 A
