# Supplementary material for: Gibberellic acid induced parthenocarpic ‘Honeycrisp’ apples (Malus domestica) exhibit reduced ovary width and lower acidity
Source: Hortic Res. 2019 Apr 6;6:41. doi: 10.1038/s41438-019-0124-8 (PMC6441655; doi:10.1038/s41438-019-0124-8)
Supplement: Supplementary file 9 — Table S1 [file 41438_2019_124_MOESM9_ESM.docx]

| Fruit Retention | | | | | |
| --- | --- | --- | --- | --- | --- |
| Treatment | 14 DAT | 28 DAT | 36 DAT | 50 DAT | 131 DAT |
| GA_3_ | 84% A | 31% A | 19% A | 14% A | 9% A |
| NAA | 80% A | 29% A | 19% A | 16% A | 0% B |
| GA_3_ + NAA | 69% A | 28% A | 21% A | 14% A | 0% B |
| NPA | 68% A | 3% B | 1% B | 0% B | 0% B |
| Negative Control | 56% A | 2% B | 2% B | 2% B | 0% B |
| Hand-pollinated Control | 67% A | 39% A | 36% C | 32% C | 16% C |
| Open-pollinated Control | 80% A | 56% C | 46% C | 44% D | 20% C |

Table S1
