# Supplementary material for: Gibberellic acid induced parthenocarpic ‘Honeycrisp’ apples (Malus domestica) exhibit reduced ovary width and lower acidity
Source: Hortic Res. 2019 Apr 6;6:41. doi: 10.1038/s41438-019-0124-8 (PMC6441655; doi:10.1038/s41438-019-0124-8)
Supplement: Supplementary file 10 — Table S2 [file 41438_2019_124_MOESM10_ESM.docx]

| Average size of fruit | | | | | |
| --- | --- | --- | --- | --- | --- |
| Treatment | 14 DAT | 28 DAT | 36 DAT | 50 DAT | 131 DAT |
| GA_3_ | 56.96 A | 323.24 A | 869.61 A | 1606.34 AD | 5026.61 A |
| NAA | 21.84 B | 27.75 B | 33.80 B | 20.25 B | NA |
| GA_3_ + NAA | 26.23 B | 34.47 B | 31.55 B | 53.12 B | NA |
| NPA | 23.78 B | 322.19 A | 542.57 C | 673.48 C | NA |
| Control | 21.23 B | 279.99 A | 354.94 C | 1340.38 A | NA |
| Hand Pollinated | 43.03 C | 445.10 A | 963.35 A | 1684.24 D | 5086.33 A |
| Open Pollinated | 42.72 C | 445.37 A | 1100.19 D | 1765.12 D | 5213.57 A |

Table S2
