# Supplementary material for: Gibberellic acid induced parthenocarpic ‘Honeycrisp’ apples (Malus domestica) exhibit reduced ovary width and lower acidity
Source: Hortic Res. 2019 Apr 6;6:41. doi: 10.1038/s41438-019-0124-8 (PMC6441655; doi:10.1038/s41438-019-0124-8)
Supplement: Supplementary file 14 — Supplementary File [file 41438_2019_124_MOESM14_ESM.docx]

**Figure S1. Apple flower and fruit morphology.** In the apple flower, the hypanthium (green) is a cup-like structure fused to the outside of the ovary (lavender). The hypanthium enlarges and forms the fleshy portion of the mature apple fruit.

**Figure S2. Extended Venn Diagrams.** Venn Diagrams from Figure 4B, with enriched pathway (bin) lists generated by MapMan using Wilcoxon Rank Sum Test with Benjamin Hochberg correction, p ≤ 0.05.

**Figure S3. Comparison of cell size in GA_3_-treated and hand-pollinated ovary and hypanthium.** **a.** Representative sections used for measuring and counting cells. Scale bar = 100µm. **b.** Quantification of average cell size, from three sections per three replicates. Identical letters indicates statistical similarity (p ≤ 0.05); significance was calculated using unpaired t-test. **c.** Total cell number, quantified from the same sections.

**Figure S4. Expression of putative apple orthologs of tomato shape genes *SUN*, *OVATE, CNR* and *KLUH*.** Two orthologs for each gene were identified, most abundant ortholog is shown on left. **a.** Average MD02G1297300 expression is significantly lower in GA_3_-treated ovule compared to hand-pollinated at 18 DAT. MD14G1079600 expression is low throughout. **b.** There are no significant differences in average MD02G1151100 expression between GA_3_-treated and hand-pollinated tissues. Expression of MD15G1030700 is low throughout. **c.** Expression of both MD10G1094000 and MD05G1079900 is low throughout. **d.** Expression of the KLUH ortholog MD13G1060500 was highest in hand-pollinated ovule while MD16G1059700 was low throughout. Significance was calculated within tissue-type using ANOVA and Tukey HSD. No statistical comparisons were done for genes with < 5 TPM. *MdSUN* and *MdOVATE* genes are putative orthologs TPM = Transcripts Per Million, scales vary.

**Figure S5. Validation of RNA-seq by qPCR.** Comparative expression of three GA biosynthesis and two MADS box genes, quantified by qPCR, RNA-seq and RNA-seq corrected by *MdTEF2.*

**Table S1. Percentage fruit retained following treatment**. Letters indicate Tukey HSD values, calculated among treatments for each date (columns). Identical letters indicate statistical similarity.

**Table S2. Size of fruit following treatment.** Values indicate average oval area, calculated at the widest, longest portion of each fruit. Letters indicate Tukey HSD values, calculated among treatments for each date (columns). Identical letters indicate statistical similarity. Asterisks indicate averages where fruit was retroactively determined to be seeded. All values are in mm^2^.

**Table S3. Enriched Mapman pathways of genes differentially expressed between GA_3_-treated and hand-pollinated tissues at 18 and 132 DAT.** p-values reported for uncorrected Wilcoxon Rank Sum Test. For the Cell Division and Hormone Metabolism pathways enriched in ovary at 18 DAT, gene IDs, orthologs identified in the description, and fold differences are listed.

**Table S4. Genes differentially expressed between GA_3_-treated and hand-pollinated hypanthium and ovary involved in Cell Division and Cell Cycle or Cell Wall modification and Cell Wall Degradation.** Pathways and descriptions identified by Mapman analysis. Summary of up and down-regulated DEGs in each category is presented.

**Table S5. Published Effects of Exogenous GA Applications on Apples.**
